# WISDOM-RELATED EMOTION REGULATION RESOURCES EXPLAIN MIDDLE-AGED AND OLDER ADULTS’ COVID-19-RELATED DISTRESS

**DOI:** 10.1093/geroni/igad104.0394

**Published:** 2023-12-21

**Authors:** Crystal Park, Dahee Kim, Beth Russell, Michael Fendrich

**Affiliations:** University of Connecticut, Storrs Mansfield, Connecticut, United States; UCONN, Storrs Manfield, Connecticut, United States; UCONN, Storrs Manfield, Connecticut, United States; UCONN, Storrs Manfield, Connecticut, United States

## Abstract

As the pandemic wrought wide-reaching disruption across the world, younger adults appeared to be faring more poorly than other adults. We hypothesized that younger adults might possess fewer emotion regulation resources and skills, accounting for their relatively high levels of distress. In data gathered from a national sample of 1258 adults, we examined how baseline resources (in mid-April, during initial peak infections) predicted distress (depression, anxiety, PTSD symptoms) five weeks later, when states began initial re-openings. Younger adults (18-35 years; n = 317; mean age = 29.2 years) reported greater distress and less social support, mindfulness, and emotion regulation skills than did middle aged (36-60 years; n = 513; mean age =51.7 years) and older adults (61-88 years; n =428; mean age = 70.1 years). Controlling for stress exposure, younger adults’ distress was predicted by impulsivity and lack of perceived strategies while middle-aged and older adults’ (lower) distress was predicted by acceptance of negative emotions and emotion regulation abilities; perceived social support was related to lower distress for all groups but mindfulness was unrelated. Results suggest that emotion regulation resources and skills are a promising prevention and intervention focus.

